# Assessment of acute stroke care, stroke metrics and patient outcomes: analysis from the pre-implementation phase of the IMPETUS stroke study

**DOI:** 10.3389/fneur.2025.1697658

**Published:** 2025-12-03

**Authors:** Shweta Gupta, Rohit Bhatia, Madakasira Vasantha Padma Srivastava, Partha Haldar, Mamta Bhushan Singh, Manish Salunkhe, Imnameren Longkumer, Deepshikha Prasad, Risha Sarkar, Vijay Sardana, Dilip Maheshwari, Bharat Bhushan, Alok Verma, Nikhil Dongre, Nikhil Sahu, Samhita Panda, Sucharita Anand, Biman Kanti Ray, Inder Puri, Paresh Zanzmera, Amit Gamit, Sanjeev Kumar Bhoi, Menka Jha, Priyanka Samal, Seepana Gopi, Garuda Butchi Raju, Amit Bhardwaj, Raminder Singh Sibia, Rupinderjeet Kaur, Ashutosh Tiwari, Niraj Kumar, Mritunjai Singh, Kiran Bala, Surekha Dabla, Mahindar Pal Singh Chawla, Jyoti Garg, Shishir Chandan, Rupali Malik, Thomas Iype, Chithra Pushpa, Ashok Kumar, Abhay Ranjan, Ravinder Garg, Sulena Sulena, Pramod Darole, Gurpreet Chhina, Shalin Shah, Sudhir Shah, Gajendra Ranga, Smita Nath, Alvee Saluja, Lekhraj Hemraj Ghotekar, Venugopalan Y. Vishnu, Roopa Rajan, Anu Gupta, Deepti Vibha, Rajesh Kumar Singh, Awadh Kishor Pandit, Ayush Agarwal, Amit Rohila, Pushpinder Khera, Sarbesh Tiwari, Suryanarayanan Bhaskar, Mayank Garg, Anupam Dey, Satyabrata Guru, Suprava Naik, T. Sateesh Kumar, Minakshi Dhar, Naman Agrawal, Mayank Patel, Pranav Joshi

**Affiliations:** 1 Department of Neurology, All India Institute of Medical Sciences, New Delhi, India; 2 Centre for Community Medicine, All India Institute of Medical Sciences, New Delhi, India; 3 Department of Neurology, Kota Medical College, Kota, India; 4 Department of Neurology, Ganesh Shankar Vidyarthi Memorial Medical College, Kanpur, India; 5 Department of Neurology, All India Institute of Medical Sciences, Jodhpur, India; 6 Department of Neurology, Bangur Institute of Neurology, IPGMER, Kolkata, India; 7 Department of Neurology, Sardar Patel Medical College, Bikaner, India; 8 Department of Neurology, Government Medical College, Surat, India; 9 Department of Medicine, Government Medical College, Surat, India; 10 Department of Neurology, All India Institute of Medical Sciences, Bhubaneswar, India; 11 Department of Neurology, Andhra Medical College, Visakhapatnam, India; 12 Department of Neurology, Dr. Rajendra Prasad Government Medical College, Tanda, India; 13 Department of Medicine, Government Medical College, Patiala, India; 14 Department of Neurology, All India Institute of Medical Sciences, Rishikesh, India; 15 Department of Neurology, Pandit Bhagwat Dayal Sharma Post Graduate Institute of Medical Sciences, Rohtak, India; 16 Department of Medicine, Atal Bihari Vajpayee Institute of Medical Sciences and Dr. Ram Manohar Lohia Hospital, New Delhi, India; 17 Department of Neurology, Atal Bihari Vajpayee Institute of Medical Sciences and Dr. Ram Manohar Lohia Hospital, New Delhi, India; 18 Department of Neurology, VMMC & Safdarjung Hospital, New Delhi, India; 19 Department of Medicine, VMMC & Safdarjung Hospital, New Delhi, India; 20 Department of Neurology, Government Medical College, Trivandrum, India; 21 Department of Neurology, Indira Gandhi Institute of Medical Sciences, Patna, India; 22 Department of Medicine, Guru Gobind Singh Medical College, Faridkot, India; 23 Department of Neurology, Guru Gobind Singh Medical College, Faridkot, India; 24 Department of Medicine, Lokmanya Tilak Municipal Medical College and General Hospital, Mumbai, India; 25 Department of Medicine, Government Medical College, Amritsar, India; 26 Department of Neurology, Sardar Vallabhbhai Patel Institute of Medical Sciences and Research, Ahmedabad, India; 27 Department of Medicine, University College of Medical Sciences, New Delhi, India; 28 Department of Neurology, Lady Hardinge Medical College, New Delhi, India; 29 Department of Medicine, Lady Hardinge Medical College, New Delhi, India; 30 Department of Medicine, All India Institute of Medical Sciences, Jodhpur, India; 31 Department of Diagnostic and Interventional Radiology, All India Institute of Medical Sciences, Jodhpur, India; 32 Department of Neurosurgery, All India Institute of Medical Sciences, Jodhpur, India; 33 Department of Medicine, All India Institute of Medical Sciences, Bhubaneswar, India; 34 Department of Emergency Medicine, All India Institute of Medical Sciences, Bhubaneswar, India; 35 Department of Radiology, All India Institute of Medical Sciences, Bhubaneswar, India; 36 Department of Medicine, All India Institute of Medical Sciences, Rishikesh, India; 37 Department of Emergency Medicine, All India Institute of Medical Sciences, Rishikesh, India

**Keywords:** stroke, infarction, intracerebral hemorrhage, implementation science, patient care, rehabilitation

## Abstract

**Background:**

Stroke is a major cause of death and disability in India. Many stroke patients seek care at government medical colleges but studies have not comprehensively assessed the quality of acute stroke care. This study aims to evaluate key indicators for optimal stroke care in the pre- implementation phase of implementation of an evaluation and treatment package for uniform stroke care (IMPETUS) study across 23 medical colleges in India.

**Methods:**

IMPETUS stroke is a multicentric, prospective, multiphase, mixed-methods, quasi-experimental implementation study, comprising three phases. During its pre-implementation phase, baseline assessment of stroke care was performed using pre-structured case report form, among prospectively enrolled acute stroke patients.

**Results:**

A total of 2,018 patients were enrolled during the pre-implementation phase. The mean (SD) age was 59.08 (14.4) years, with male preponderance (64.2%); 69.06% had an onset <24 h, majority had ischemic stroke (60.1%), followed by intracerebral hemorrhage (38.4%). Key risk factors were hypertension, diabetes, smoking, alcohol and previous stroke. Imaging performed included non-contrast computed tomography (NCCT) (69.6%), computed tomography angiography (CTA) (25.6%) and magnetic resonance angiography (MRA) (24.6%). Intravenous thrombolysis (IVT) was administered in 39.2% eligible patients, predominantly with tissue plasminogen activator (tPA) (72%). In-hospital delay was the most common reason for not receiving thrombolysis (44.8%). The median door-to-CT, CT-to-needle and door-to-needle time were 95, 36.5 and 67 min, respectively. Other important stroke care indices were also evaluated. In-hospital mortality was 19.4 and 33.1% patients achieved modified rankin scale (mRS) score 0–2 at 90-days.

**Conclusion:**

This comprehensive data provides a representative baseline status of acute stroke care in select medical colleges across India, which will be useful in comparing advancements during the implementation phase and improve policy making.

## Introduction

Worldwide, stroke has emerged as the second leading cause of death and the third leading cause of combined death and disability (as expressed by disability-adjusted life-years lost-DALYs) according to the most recent Global Burden of Disease (GBD) 2021 estimates (1). There has been a substantial increase in the incidence of stroke with low-income and lower-middle-income countries (LMICs) carrying the largest share of the global stroke burden. India accounts for 13.3% of the global disability-adjusted life years (DALYs) lost due to stroke with a relatively younger age of onset compared to the western population (2).

Stroke care is not uniform across both public and private sectors and could be related to several factors including felt need, infrastructural deficiency, limited trained or experienced manpower and administrative support (3). While a national program for stroke (4) and established stroke management guidelines exist (5), the delivery of organized stroke care continues to face significant challenges in India. Medical colleges, as integral components of the public health system, function as essential links between rural, district, and other tertiary levels of care centres (6). However, in the absence of a systematic performance evaluations, the quality and outcomes of stroke care provided are often presumed to be optimal, rather than being substantiated by empirical evidence. To date, there are no studies from India that comprehensively assessed the quality of acute stroke care.

The implementation of an evaluation and treatment package for uniform stroke care across medical colleges of India (IMPETUS stroke) study (3) was an implementation research study conducted between October 2021 to December 2024 with the aim to improve stroke care across 23 medical colleges from the time of stroke recognition in the emergency, inpatient management (admitted patients), secondary stroke prevention, and appropriate discharge planning. This study is one of the first prospective, multicenter evaluations focused specifically on public-sector tertiary medical colleges. It provides a detailed, real-world snapshot of stroke care across a wide range of settings before the implementation of any standardized intervention. The objectives of the present study were to assess the status of stroke care observed during the pre-implementation phase of the IMPETUS stroke study across all collaborating centres. It aims to evaluate the existing stroke care practices, infrastructure and documentation processes across all collaborating centres. Findings from this phase would help evaluate the improvement, impact and sustainability of the implementation in the stroke care pathway in subsequent phases.

## Methods

### Study design

IMPETUS stroke was a multicentric, prospective, multiphase, mixed-methods, quasi-experimental implementation study intended to examine changes in a select set of acute stroke care-related indicators over time within sites exposed to the same implementation strategy (3). It comprised three phases: Phase I (pre-implementation), Phase II (implementation) and Phase III (post implementation). The study was initiated in October 2021. Phase I was the pre-implementation phase, expanded from a period of 3–5 months duration, wherein a baseline assessment of existing stroke care components was made. Quantitative data were collected from patients using a pre-defined structured form to assess stroke care components. Patient outcomes at the end of 3 months were abstracted from in-person follow-up, tele-communication or medical records. Focus group discussions were also held to understand barriers and facilitators of stroke care.

### Study settings

The study was conducted at 23 collaborating medical colleges and affiliated public hospitals stretched across 14 different cities in 12 different states of India. The study was approved by the Institute Ethics Committee, All India Institute of Medical Sciences (AIIMS), New Delhi, India (Reference number: IEC-92/06.3.2020) and respective ethics committees of all collaborating colleges.

### Study participants

All consecutive patients with acute stroke within 72 h of onset [ischemic stroke (IS), intracerebral hemorrhage (ICH) and cerebral venous sinus thrombosis (CVST)] admitted to the emergency or inpatients units were recruited after obtaining informed consent. Caregivers of patients with stroke (those who spend at least 6 h per day with the patient) were also recruited. Paid professional caregivers were not eligible to participate in the study.

### Study tool

The authors designed the case record form based on a predefined checklist that included: (1) Baseline admission details, (2) Thrombolysis and thrombectomy data, (3) Laboratory details, (4) In hospital details up to 72 h, (4) Discharge details, (5) Caregiver Knowledge assessment, (6) Follow-up information. This quantitatively assessed different parameters of patient care. The Redcap database^1^ at AIIMS, Delhi was used to enter the details from each collaborating site.

### Study outcomes

The present study assessed the status of stroke care on various parameters observed during the specific period of the IMPETUS stroke study. Three months’ disability outcome was assessed using a modified Rankin Scale (mRS) score dichotomized as ≤2 as a good outcome.

### Data availability and access

The data are available upon reasonable request from the corresponding author.

### Statistical analysis

Descriptive statistics summarized patient demographic and clinical characteristics using mean (standard deviation), median (interquartile range) or frequency (percentage), as appropriate. Characteristics were stratified according to type of stroke [ischemic stroke (IS), intracerebral hemorrhage (ICH) and cerebral venous sinus thrombosis (CVST)] and differences between groups were assessed using chi-square test and student’s t-test as appropriate. A *p*-value <0.05 was considered statistically significant. STATA version 14.1 was used to perform all data analyses.

## Results

### Demographic characteristics

Overall, a total of 2,018 patients were recruited during the pre-implementation phase of the study across all participating medical centres, with a mean (SD) age of 59.08 (14.4) years. Among these, 64.2% were males and 35.8% were females. Patients presenting with an onset of symptoms within 24 h comprised 69.06% whereas 18.39 and 12.54% of patients presented with symptom onset within 24–48 and 48–72 h, respectively. The majority of patients presented with ischemic stroke (60.11%), followed by intracerebral hemorrhage (38.40%) and cerebral venous sinus thrombosis (CVST) (1.49%). The National Institute of Health Stroke Scale (NIHSS) at admission was recorded in 20.33% of patients. Vital monitoring in the emergency setting, such as blood pressure and blood sugar was measured in 97.07 and 63.16%, respectively, (Table 1). Hypertension was the most common major risk factor observed in 80.71% of patients. Other risk factors included diabetes (30.34%), smoking (22.36%), alcohol consumption (24.05%), coronary artery disease (9.22%), rheumatic heart disease (3.02%), atrial fibrillation (4.76%), dyslipidemia (3.62%), previous stroke (21.17%), and family history of stroke among 12.79% of the cases (Table 1).

**Table 1:** Admission characteristics of overall patients.

Variables	Overall *n* = 2,018	Ischemic Stroke *n* = 1,213 (60.11%)	Intracerebral Hemorrhage *n* = 775 (38.40%)	CVST *n* = 30 (1.49%)	*p*-value
Age^†^ (years), mean (SD)	59.08 (14.43)	60.85 (14.12)	57.01 (14.09)	41.20 (17.08)	<0.001
Gender^†^					
Males	1,295 (64.20)	764 (63.04)	511 (65.94)	20 (66.67)	0.405
Females	722 (35.80)	448 (36.96)	264 (34.06)	10 (33.33)	
Onset of Symptoms^†^					
<24 Hours	1,393 (69.06)	837 (69.00)	537 (69.38)	19 (63.33)	0.802
24–48 Hours	371 (18.39)	223 (18.38)	143 (18.48)	5 (16.67)	
48–72 Hours	253 (12.54)	153 (12.61)	94 (12.14)	6 (20.00)	
Whether time of onset is recorded^†^ (Yes)	1,075 (53.30)	695 (57.30)	363 (46.90)	17 (56.67)	<0.001
Whether baseline NIHSS is recorded at admission^†^ (Yes)	410 (20.33)	333 (27.45)	73 (9.43)	4 (13.33)	<0.001
Whether baseline BP is measured in the emergency^†^ (Yes)	1958 (97.07)	1,180 (97.28)	748 (96.64)	30 (100.00)	0.450
Whether baseline blood sugar monitored at admission^†^ (Yes)	1,274 (63.16)	810 (66.78)	442 (57.11)	22 (73.33)	<0.001
Risk factors					
Hypertension^**†**^	1,628 (80.71)	909 (74.94)	708 (91.47)	11 (36.67)	<0.001
Diabetes^†^	612 (30.34)	431 (35.53)	179 (23.13)	2 (6.67)	<0.001
Smoking^†^	451 (22.36)	293 (24.15)	150 (19.38)	8 (26.67)	0.122
Alcohol Consumption^†^	485 (24.05)	265 (21.85)	210 (27.13)	10 (33.33)	0.013
Coronary Artery Disease (CAD)^†^	183 (9.22)	143 (11.79)	42 (5.43)	1 (3.33)	<0.001
Rheumatic Heart Disease (RHD)^†^	61 (3.02)	49 (4.04)	12 (1.55)	0 (0.00)	0.004
Atrial Fibrillation (AF)^†^	96 (4.76)	65 (5.36)	29 (3.75)	2 (6.67)	0.229
Dyslipidemia^†^	73 (3.62)	44 (3.63)	29 (3.75)	0 (0.00)	0.559
Any Previous History of Stroke^†^	427 (21.17)	286 (23.58)	139 (17.96)	2 (6.67)	0.002
Any Family History of Stroke^†^	258 (12.79)	123 (10.14)	131 (16.93)	4 (13.33)	<0.001

Data are presented as *n* (%) or mean (standard deviation). CVST, cerebral venous sinus thrombosis; NIHSS, National Institute of Health Stroke Scale; BP, Blood pressure. Variables with “Yes” in parentheses indicate those that responded “yes” out of the total patients. The corresponding number and per cent of “no” response is not indicated in the table.

† Missing data: *n* = 2 (age); *n* = 1 (gender, onset of symptoms, time of onset recorded, baseline NIHSS recorded at admission, baseline BP measured in the emergency, baseline sugar monitored at admission, hypertension, diabetes, smoking, alcohol consumption, coronary artery disease, rheumatic heart disease, atrial fibrillation, dyslipidemia, any previous history of stroke, any family history of stroke).

### Imaging and acute stroke treatment

Non-contrast computed tomography (NCCT) was performed in 69.67% of patients at admission (Table 2). In many centres, other imaging facilities such as computed tomography angiography (CTA) (73.51%), magnetic resonance angiography (MRA) (73.57%) and doppler neck vessels (52.97%) were not performed when indicated due to various reasons.

**Table 2:** Imaging characteristics of admitted patients.

Variables	*n* (%)
NCCT at admission (*n* = 2018)	
Yes	1,406 (69.67)
No	154 (7.63)
Done Outside of study centre	458 (22.70)
CT Angiography (*n* = 2017)^†^	
Indicated^*^	1,431 (70.95)
Not indicated	586 (29.05)
CT Angiography done among indicated patients (*n* = 1,431)	
Yes	367 (25.65)
No	1,052 (73.51)
Not available	12 (0.84)
MR Angiography (*n* = 2017)^†^	
Indicated^*^	1,188 (58.90)
Not indicated	829 (41.10)
MR Angiography done among indicated patients (*n* = 1,188)	
Yes	293 (24.66)
No	874 (73.57)
Not available	21 (1.78)
Doppler Neck vessels (*n* = 2016)^†^	
Indicated^*^	978 (48.51)
Not indicated	1,038 (51.49)
Doppler Neck vessels done among indicated patients (*n* = 978)	
Yes	420 (42.94)
No	518 (52.97)
Not available	40 (4.09)

NCCT, non-contrast computed tomography; CT, computed tomography; MR, magnetic resonance.

† Missing data: *n* = 2 (doppler neck vessel), *n* = 1 (CT angiography, MR angiography).

* CTA/MRA/Doppler was “indicated” in patients diagnosed with ischemic stroke as a standard evaluation for assessment of vascular status of both extracranial and intracranial arteries to classify the etiology of stroke using TOAST classification and also look for presence of large artery occlusion in the acute phase to assess eligibility for EVT.

Among ischemic stroke patients eligible for thrombolysis (15.75%), intravenous thrombolysis (IVT) was administered in 39.27% (Table 3). Tissue plasminogen activator (tPA) was the most common agent used for IVT. In-hospital delay after admission (44.83%) was one major reason for patients not being thrombolysed (Table 3), followed by non-availability of IVT (28.45%), minor stroke (12.07%), negative consent (8.62%), and unaffordability (6.03%). Endovascular therapy (EVT) was provided to only 1.19% of patients as most centres did not have EVT services (54.8%). Several other reasons for exclusion are also mentioned in Table 3. Time-sensitive quality measures showed that the median duration from symptom onset to hospital admission was 660 min (IQR 285–1,682). The overall median door-to-CT scan time was 95 min (IQR 46–274), with ischemic stroke patients having a shorter imaging time of 87 min (IQR 44–242) compared to 120 min (IQR 53–369) for intracerebral hemorrhage (ICH).

**Table 3:** Acute management and stroke metrics among ischemic stroke patients.

Variables	*n* (%)/median (IQR)
Patients eligible for thrombolysis (out of total 1,213 IS patients)^§^	191 (15.75)
≤60 min	30 (15.71)
60–180 min	91 (47.64)
180–270 min	59 (30.89)
>270 min	11 (5.76)
Patients thrombolysed (out of 191 eligible patients)	75 (39.27)
≤60 min	10 (13.33)
60–180 min	47 (62.67)
180–270 min	13 (17.33)
>270 min	5 (6.67)
Type of thrombolysis given (*n* = 75)	
tPA	54 (72.00)
TNK	21 (28.00)
Reasons for eligible patients not being thrombolysed (116 out of 191 patients eligible for thrombolysis)	
Thrombolysis not available	33 (28.45)
Thrombolysis not affordable	7 (6.03)
Available but not given^*^	14 (12.07)
In-hospital delay after admission	52 (44.83)
Refused	10 (8.62)
EVT performed (out of 837 IS patients with time of onset <24 h)	10 (1.19)
Reasons for EVT not performed on eligible patients^#^ (out of 1,203 IS patients^†^)	
No LVO	60 (5.0)
EVT Not available	660 (54.8)
EVT Not Affordable	12 (1.0)
EVT Available but Not used	14 (1.2)
Not Eligible (time window)	336 (27.9)
Not eligible (contraindications)	105 (8.7)
Refused	16 (1.3)
Time sensitive quality metrics, median (IQR)	
Onset-to-Door Time: Overall patients (*n* = 1774)	660 (285–1,682)
Ischemic Stroke (*n* = 1,046)	647.5 (280–1,624)
ICH (*n* = 703)	690 (287–1746)
CVT (*n* = 25)	630 (300–1,465)
Door-to-CT scanner time: Overall patients (*n* = 1,406)	95 (46–274)
Ischemic Stroke (*n* = 911)	87 (44–242)
ICH (*n* = 479)	120 (53–369)
CVT (*n* = 16)	140.5 (41.5–512.5)
CT scanner-to-Needle time among thrombolysed patients (*n* = 70^‡^)	36.5 (23–50)
Onset-to-Needle time among thrombolysed patients (*n* = 70^‡^)	201.5 (165–250)
Door-to-Needle time among thrombolysed patients (*n* = 75)	67 (48–90)

Data are presented as *n* (%) or median (interquartile range [IQR]); IS, Ischemic Stroke; ICH, Intracerebral Hemorrhage; tPA, Tissue Plasminogen Activator; TNK, Tenecteplase; EVT, Endovascular therapy (Mechanical Thrombectomy); LVO, Large vessel occlusion; CT, computed tomography.

* Patients presented with minor stroke (NIHSS ≤5 at presentation).

‡ Data available for 70 patients out of 75 thrombolysed patients.

† *n* = 10 missing data on EVT among 1,213 IS patients.

§ IVT eligibility: Ischemic stroke presenting within ≤4.5-h window and eligible for thrombolysis by standard guidelines.

# EVT eligibility: Confirmed large vessel occlusion (LVO), within ≤6 h of window or up to 24 h based on imaging criteria.

Among stroke patients eligible for thrombolysis, the onset-to-needle time was 201.5 min (IQR 165–250), the door-to-needle time was 67 min (IQR 48–90), and the CT-to-needle time was 36.5 min (IQR 23–50) (Table 3). A detailed presentation of between-site variation for both door-to-CT time and intravenous thrombolysis (IVT) use is provided in Supplementary Table 2.

### In hospital stroke care management

During hospital admission, patient-level information for three consecutive days (up to 72 h) were collected to correlate stroke care practices with patient outcomes. Body temperature and blood pressure were routinely assessed in all centres as compared to assessment of level of consciousness (GCS) and blood glucose monitoring (Table 4). Routine swallow assessments were performed in 55.6, 40.93 and 41.75% on day 1, 2 and 3, respectively. It was observed that in 44.4% of patients, routine assessments were not done on day 1, and around 39.39% were marked as non-eligible based on their level of consciousness. As per the patient’s mobility status, deep vein thrombosis (DVT) prophylaxis was provided to 20.9% on day 1; it was not indicated in the majority (53.74%) and 79.10% of the eligible patients did not receive it. Intermittent pneumatic compression devices (49.48%) was most commonly used, followed by heparin (41.24%) and a combination of both (9.28%). Further details for day 2 and day 3 are reported in Table 4. Physiotherapy consultation was performed in 56.4, 69.59 and 74.25% on day 1, 2, and 3, respectively. At centres where rehabilitation personnel were not available or insufficient, physiotherapy consultation was not provided to eligible patients (43.6, 30.41 and 25.75%, respectively). Similarly, air mattresses were not provided to 39.76% on day 1 due to their unavailability and were only given to 37.37, 40.65 and 42.74% on consecutive days. However, most patients received appropriate positioning (70.48, 75.70 and 78.95% on day 1, 2 and 3, respectively) during hospital stay. Stroke care advice was provided to caregivers in 69.98% of cases on day 3. Overall, complications occurred in low proportions (4.49, 5.99 and 7.6% each day).

**Table 4:** In-hospital stroke care components.

Variables	Day 1 (*n* = 2006^*^)	Day-2 (*n* = 1822^*^)	Day-3 (*n* = 1659^*^)
Vital monitoring			
Whether GCS recorded (Yes)	1,235 (61.57)	851 (46.71)	733 (44.18)
Whether temperature monitored^‡^ (Yes)	1,690 (84.25)	1,516 (83.30)	1,379 (83.12)
Whether BP monitored (Yes)	1,528 (76.17)	1,462 (80.24)	1,307 (78.78)
Whether sugar monitored^§^ (Yes)	1,053 (52.49)	805 (44.21)	693 (41.87)
Secondary prevention			
Swallow assessment^†^			
Indicated	1,214 (60.61)	860 (47.20)	752 (45.33)
Not indicated	789 (39.39)	962 (52.80)	907 (54.67)
Swallow assessment performed among indicated patients (*n* = 1,214 in day-1, *n* = 860 in day-2, *n* = 752 in day-3)			
Yes	675 (55.60)	352 (40.93)	314 (41.75)
No	539 (44.40)	508 (59.07)	438 (58.25)
Deep Vein Thrombosis (DVT) prophylaxis			
Indicated	928 (46.26)	819 (44.95)	715 (42.98)
Not indicated	1,078 (53.74)	1,003 (55.05)	946 (57.02)
DVT Prophylaxis received among indicated patients (*n* = 928 in day-1, *n* = 819 in day-2, *n* = 715 in day-3)			
Yes	194 (20.90)	207 (25.28)	204 (28.61)
No	734 (79.10)	612 (74.72)	509 (71.39)
Types of DVT prophylaxis among those who received it (*n* = 194 in day-1, *n* = 207 in day-2, *n* = 204 in day-3)			
Heparin/LMWH	80 (41.24)	79 (38.16)	76 (37.25)
Compression device	96 (49.48)	100 (48.31)	99 (48.53)
Both	18 (9.28)	28 (13.53)	29 (14.22)
Physiotherapy consultation			
Indicated	1,640 (81.75)	1,565 (85.89)	1,433 (86.38)
Not indicated	366 (18.25)	257 (14.11)	226 (13.62)
Physiotherapy consultation received among indicated patients (*n* = 1,640 in day-1, *n* = 1,565 in day-2, *n* = 1,433 in day-3)			
Yes	925 (56.40)	1,089 (69.59)	1,064 (74.25)
No	715 (43.60)	476 (30.41)	369 (25.75)
Air mattress			
Indicated	1,504 (74.98)	1,375 (75. 47)	1,240 (74.74)
Not indicated	502 (25.02)	447 (24.53)	419 (25.26)
Air mattress received among indicated patients (*n* = 1,504 in day-1, *n* = 1,375 in day-2, *n* = 1,240 in day-3)			
Yes	562 (37.37)	559 (40.65)	530 (42.74)
No	344 (22.87)	307 (22.33)	269 (21.69)
Not available	598 (39.76)	509 (37.02)	441 (35.57)
Appropriate positioning^‡^			
Indicated	1,687 (84.10)	1,531 (84.07)	1,392 (83.91)
Not indicated	319 (15.90)	290 (15.93)	267 (16.09)
Appropriate positioning received among indicated patients (*n* = 1,687 in day-1, *n* = 1,531 in day-2, *n* = 1,392 in day-3)			
Yes	1,189 (70.48)	1,159 (75.70)	1,099 (78.95)
No	498 (29.52)	372 (24.30)	293 (21.05)
Whether caregiver advice is given (Yes)	1,353 (67.45)	1,266 (69.48)	1,161 (69.98)
Whether in-hospital complications are recorded^‡ §^ (Yes)	90 (4.49)	109 (5.99)	126 (7.60)

Data are presented as *n* (%) GCS, Glasgow coma scale; BP, Blood pressure; LMWH, Low molecular weight heparin. Variables with Yes in parenthesis indicate those that responded ‘yes’ out of the total patients on each day. The corresponding number and per cent of ‘no’ responses are not indicated in the table.

* As per the duration of the stay in the hospital.

† Missing data in day-1: *n* = 3 (swallow assessment).

‡ Missing data in day-2: *n* = 1 (whether sugar is monitored, appropriate positioning, whether in-hospital complications are recorded), *n* = 2 (whether temperature monitored).

§ Missing data in day-3: *n* = 4 (whether sugar monitored), *n* = 1 (whether in-hospital complications are recorded).

### Discharge status and secondary prevention

Overall, in-hospital mortality was observed in 388 (19.4%) patients. A total of 304 (18.86%) patients left or were discharged against medical advice (LAMA/DAMA) among survivors (*n* = 1,612; 81.14%). At discharge, in-hospital complications were recorded in 11.2% of patients. TOAST criteria for etiological identification were missing in the majority (82.4%) of ischemic stroke patients. Large artery atherosclerosis (41.72%) was the most common etiological factor based on the evaluation (Supplementary Table 1). Among hypertensive patients, 42.66% received risk factor advice at discharge. Advice for other risk factors such as diabetes and dyslipidemia management was given in 17.05 and 8.25%, respectively. Patient care advice regarding tracheostomy (9.23%), catheter care (22.75%), Ryle’s tube feeding (28.69%) and positioning (35.95%) was documented in discharge summaries. However, care advice was not documented when indicated on the discharge summary. Advice on medication adherence (37.39%), medication dose (97.7%), timing (91.45%) and adverse effects (18.35%) was provided and documented in the discharge summary. Caregivers of 68.02% of patients were counselled and properly advised. Follow-up advice regarding when to follow up (86.94%), whom to follow up (62.44%) and where to follow up (85.87%) were mentioned in discharge summaries.

### mRS at 3 months

**Figure 1:**
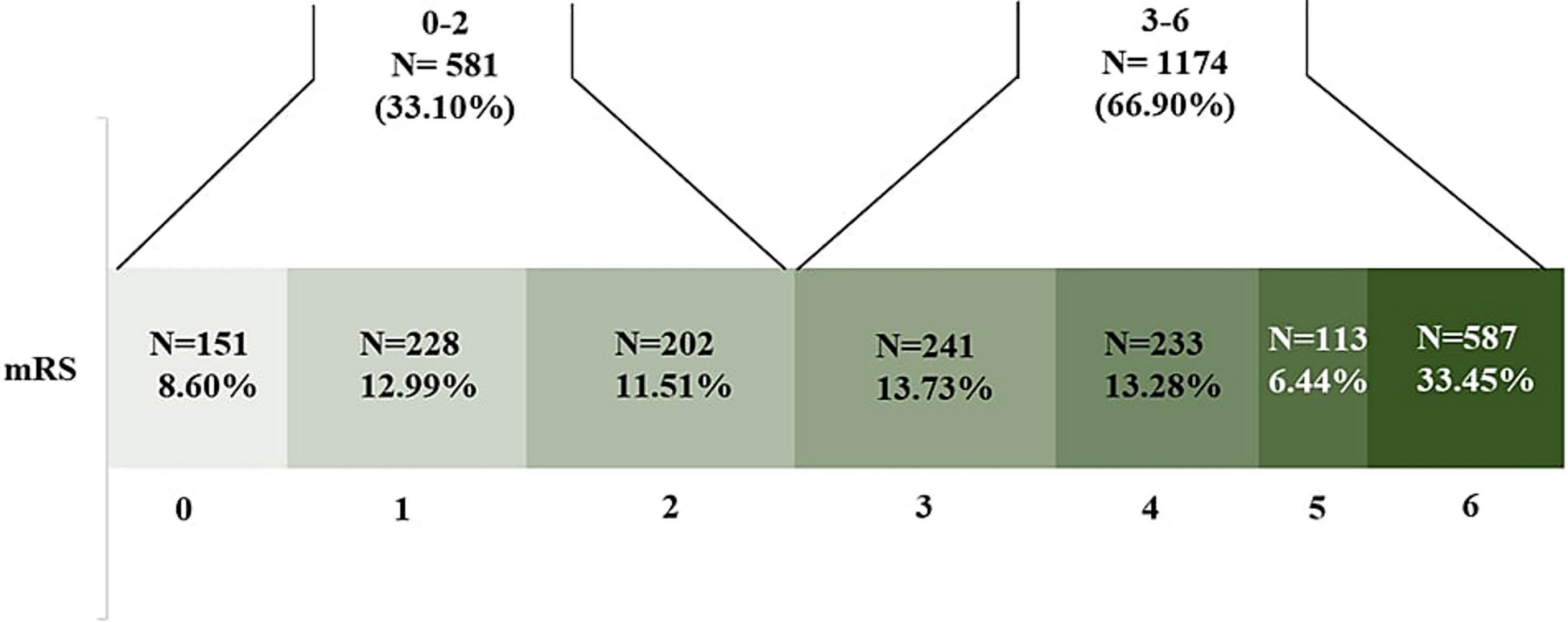
Distribution (n [%]) of modified Rankin Scale (mRS) scores at 90-days follow-up in the overall cohort (*n* = 1755), showing the proportion of patients achieving good outcome (mRS 0–2, *n* = 581 [33.10%]) versus poor outcome (mRS 3–6, *n* = 1,174 [66.90%]).

**Figure 2:**
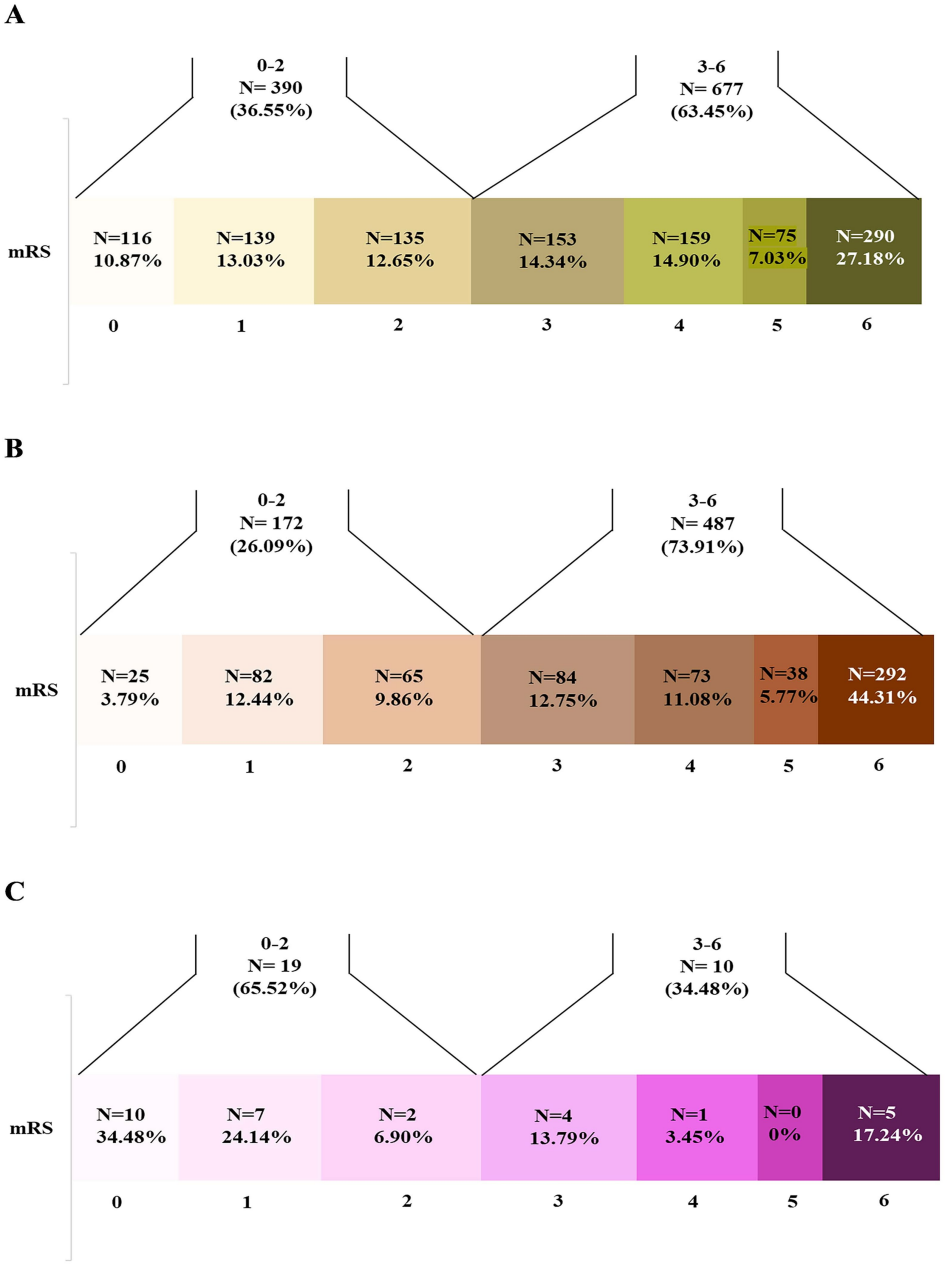
Distribution (n [%]) of modified Rankin Scale (mRS) scores at 90-days follow-up in stroke subtypes (total 1,067 ischemic stroke, 659 intracerebral hemorrhage, and 29 cerebral venous thrombosis patients). Patients with good (mRS 0–2) versus poor outcomes (mRS 3–6) varied across subtypes: **(A)** Ischemic stroke (*n* = 390 [36.55%] vs. *n* = 677 [63.45%], respectively); **(B)** Intracerebral hemorrhage (*n* = 172 [26.09%] vs. *n* = 487 [73.91%], respectively); **(C)** Cerebral venous thrombosis (*n* = 19 [65.52%] vs. *n* = 10 [34.48%], respectively).

Overall, 33.1% of patients achieved a modified Rankin Scale (mRS) score of 0–2 at 90-day follow-up: 36.55% in ischemic stroke (IS), 26.09% in intracerebral hemorrhage (ICH) and 65.52% in cerebral venous sinus thrombosis (CVST) patients, respectively (Figures 1, 2).

## Discussion

The present study highlights the status of stroke care among collaborating centres of the IMPETUS stroke implementation study (3, 7) in its pre implementation phase and the potential gaps in the stroke care pathway. This also helps provide real time information on the various key variables that impact stroke care and thereby its outcome. One of the critical challenges in stroke management is the treatment gap in public healthcare settings. The available data reflects the status across the stroke care continuum, rapid access, acute stroke treatment and care, discharge and secondary prevention.

Population level studies from lower-middle-income countries (LMICs) reported higher incidence of stroke and probable increase in the future stroke rates (8, 9). In the INTERSTROKE study of 12,342 patients with stroke from 108 hospitals in 28 countries, individuals in LMICs more often had severe strokes, intracranial hemorrhages, poorer access to services, and fewer investigations and treatments than those in Higher income countries (HICs) (10).

We observed a large proportion of patients arriving at the hospital after 24 h of stroke onset. Lack of public awareness about the warning signs of stroke symptoms, sociocultural beliefs and transport accessibility leads to lengthy delays in presenting at the hospital (11, 12). Timely recognition of stroke onset is crucial for determining a patient’s eligibility for time dependent acute ischemic stroke treatments. Previous study in a tertiary stroke centre from Romania observed that most of the stroke patients arriving after 24 h from onset were living alone and living in rural areas and highlighted the need of stroke awareness program and pre-hospital protocols (13).

Availability and accessibility of the 24/7 functional CT scan or MRI facility should be mandatory as per the national guidelines for stroke prevention and management (5). CT / MR Angiography was performed in a limited proportion of patients. Routine vascular imaging is generally not performed in many centres as surveyed by us previously (7). It may be due to issues with availability, manpower, or a general practice pattern. The absence of easy and timely access to brain imaging adds to the difficulties in diagnosing stroke in LMICs. In Ghana, only two-thirds of hospitals have a functional CT scanner available during working hours on weekdays (14). A systematic review of stroke services in Africa showed that only 13–36% of patients underwent CT or MRI scans due to its unavailability and financial constraints (15). As per guidelines recommended by healthcare professionals from the Indian Stroke Association, and other International stroke guidelines, stroke centres should be capable of performing imaging within 30 min of a patient presenting at the centre (16). In the present study, door-to-CT time exceeded the ideal threshold with an overall median CT time of 95 min. Single call notification or stroke code can significantly help in rapid evaluation of stroke patients (17). The response time towards a patient in the emergency may also vary depending upon the perceived “eligible” patient for thrombolysis and delays could happen due to other sick patients being prioritized in an extremely busy emergency in public hospitals.

The National Institute of Health Stroke Scale (NIHSS) is the most reliable and commonly used stroke severity tool in clinical trials for the administration of thrombolytic drugs (18, 19). A small proportion of baseline NIHSS assessment was observed. It is likely due to lack of training, awareness, stroke trained physicians or nurses and overburdened healthcare settings. At most centres, the medicine resident is the first contact, and not a stroke physician. The low rate of NIHSS documentation and delayed imaging likely reflects deeper system-level issues, such as the absence of standardized triage pathways, poor coordination between emergency, radiology, and neurology services, and lack of structured prehospital care. Although regular training and education may reinforce improvement, there are challenges that go beyond individual training and require broader organizational and process-level changes tailored to the Indian public health system.

Intravenous thrombolysis (IVT) and mechanical thrombectomy are an effective and approved treatment for the acute stroke management, however, their use remains limited in LMICs (20, 21). The present study observed a small proportion of ischemic stroke patients eligible for thrombolysis and within them, only one third were thrombolysed. In-hospital delay after admission and unavailability of the thrombolytic drug were observed as the most common reasons for eligible patients not being thrombolysed. A prospective study from a tertiary care hospital reported pre-hospital delay (81.5%), crowded emergency (77.7%), financial constraints (76.7%) and delay in CT scan (61.4%) as barriers to thrombolysis (22). In Peru, only 2% of patients received thrombolysis due to its unavailability. Similar observations were found for endovascular thrombectomy (EVT) (23). In our previous study of infrastructural assessment, EVT services were only available in 27% of the hospitals and only two hospitals were providing it free of cost (7). Another survey from Ghana revealed that none of the 11 major hospitals in Ghana were conducting the EVT procedure (14). Limited trained professionals, high cost and infrastructural demands limit the use of EVT in LMICs. In a survey conducted by the mission thrombectomy 2020 plus global network among 75 countries, global mechanical thrombectomy use was poor and LMICs had 88% lower mechanical thrombectomy access when compared to higher income countries (HICs) (24). Treatments such as thrombolysis and EVT should get support from national and organizational levels to become accessible at low cost or be government funded.

For the implementation of an organized system of stroke care, in-hospital management should have key elements such as stroke units, blood pressure and cardiac monitoring, Glasgow coma scale (GCS) recording, regular temperature and sugar monitoring, routine swallow assessment and interventions for prevention of secondary complications. Stroke unit admissions is one of the most effective ways to reduce hospital mortality and morbidity but its implementation still remains a challenge in LMICs (25, 26). In India, there were only 35 stroke units in 2013 with the majority in the private sector (27). Infrastructural reorganization to create a designated geographical stroke unit with 4–6 beds and training of healthcare professionals should be emphasized in low resource settings.

The present study found inconsistency in swallow assessment as nearly 27% patients did not undergo swallow evaluation during the first 24 h despite being eligible. Implementation of standardized protocols for clinical monitoring and management of temperature, sugar and swallowing assessment are highly beneficial (28). In a randomized control trial from a tertiary care center from India, reduction in the hospital mortality was reported with a nurses-led fever, sugar and swallowing bundle care (29). Data from 64 hospitals from 17 countries across Europe showed improvements in pre-to-post implementation of all three components in both high and low resource settings (30). Mandatory implementation of the protocol and frequent training for the nursing officers should be encouraged. Simple interventions such as positioning, provision of air mattress, deep vein thrombosis (DVT) prophylaxis and mobility assessments can help to reduce hospital complications and improve patient outcomes. Unavailability of the resources and a multidisciplinary team is a major barrier in providing optimum care for stroke patients.

Early mobilization and rehabilitation provide better outcomes for stroke survivors (16, 26). In this study, physiotherapy consultation was not done for almost one fourth of the patients. Most of the patients received rehabilitation advice as per consultation request. Lack of rehabilitation specialist, policies or guidelines, less prioritization, overburdened wards, lack of dedicated stroke and step-down in-patient rehabilitation units lead to compromised rehabilitation services in hospitals. Among a cohort of 250 stroke patients from Zambia, only 27% patients received physical therapy evaluation during hospitalization. Occupational and speech therapists were entirely unavailable. Similar findings had been found in mean number of in-hospital physiotherapy session (two sessions per 8-day length of stay (LOS) in Rwanda, three sessions per 12-day LOS in Tanzania, and two sessions per 7-day LOS in South Africa) (31, 32). During the INTERSTROKE study, it has been found that 77% of patients with stroke in LMICs have moderate to severe functional disability after stroke, compared with 63% in upper-middle-income countries, and 38% in HICs (10). In response to the substantial gap between the need for rehabilitation and the capacity of countries to respond to that need, the World Health Organization launched Rehabilitation 2030: A Call to Action (33). Family caregivers are an important part of stroke rehabilitation in low-resource settings (34). We observed that only a small proportion of caregivers were counselled at the time of discharge for post stroke care. Family led Rehabilitation in India and a nurse-plus-caregiver strategy in Mexico found that task shifting rehabilitation is feasible and does not jeopardize stroke survivors health (35, 36). Post discharge rehabilitation services in LMICs (31%) are much worse as compared to the HICs (92%) (37). Incorporating structured educational programs to strengthen caregiver knowledge has proven beneficial in reducing the complications both during hospitalization as well as after discharge at home (38). Training sessions using stroke manuals and video modules will be conducted during the implementation phase of this study in order to enhance caregiver knowledge.

Different stroke models can be adapted to combat these challenges within the healthcare systems of LMICs. Multidisciplinary team care has been identified as a key component in effective stroke care (39). In this system, patients with stroke are immediately identified and preferably managed in a separate stroke unit. Training and education sessions for the non-neurologist can be conducted to identify stroke symptoms in the emergency (40). Another model that was proven effective is the hub and spoke model for the timely and effective management of the stroke patient (41). Studies from India and Brazil have reported on the use of tele-stroke services (42, 43). Tele-stroke using smart phone based services can help to address the shortage of neurologists, especially in hard to reach areas and areas in which the population is highly dispersed (44).

### Strengths and limitations

The strength of this study includes its prospective, real world observational data which provide valuable insights into the current status of stroke care on various indicators that are essential and needs improvement for optimum stroke care. The current data reflect the existing system of stroke management and can help in the decision making of policies for better stroke care. This can also help to strengthen skilled and trained manpower in medical colleges to impart best available stroke care to patients. Any change or improvement in the stroke care pathway will be measurable following implementation and training phases of the IMPETUS study.

Limitations include that the data were collected from the different medical colleges in urban settings only, and economic disparities are likely to exist.

## Conclusion

This study provides the current status of stroke care in different medical colleges in India. The comprehensive data offers a representative baseline status of acute stroke management, which will be useful in assessing improvement following the intervention. Implementation of existing guidelines, quality improvement initiatives, increased number of stroke units and stroke ready hospitals, mandatory stroke orientation programs, infrastructural re-organization and capacity building will help in implementation of uniform stroke care pathway and improve stroke outcomes.

## Data Availability

The datasets presented in this article are not readily available because it is derived from a larger dataset. Requests to access the datasets should be directed to impetus.stroke@gmail.com.

## Footnotes

1
https://project-redcap.org/
