# Supplementary material for: Assessment of acute stroke care, stroke metrics and patient outcomes: analysis from the pre-implementation phase of the IMPETUS stroke study
Source: Front Neurol. 2025 Dec 3;16:1697658. doi: 10.3389/fneur.2025.1697658 (PMC12708288; doi:10.3389/fneur.2025.1697658)
Supplement: Supplementary file 1 [file Data_Sheet_1.PDF]

Supplementary Table 1: Discharge Status and Secondary Prevention

	**n (%)**
**In hospital mortality**	
a) Yes	388 (19.40)
b) No	1612 (80.60)
**Discharge status (excluding in-hospital mortality) (n=1612)**	
a) Discharge	1308 (81.14)
b) LAMA	304 (18.86)
**In hospital complications recorded at discharge (n=1696)**	190 (11.20)
Etiology Identification as per TOAST criteria (n=1309)	
Indicated	858 (65.55)
Not indicated	451 (34.45)
**Etiology Identification as per TOAST criteria among indicated (n=858)**	
a) Yes	151 (17.60)
b) No	707 (82.40)
**TOAST etiology identified**	
a) Large artery disease	63 (41.72)
b) Small vessel	14 (9.27)
c) Cardio-embolism	51 (33.77)

d) Other determined<br>e) Undetermined	4 (2.65)<br>19 (12.58)
**Risk Profiling at the time of discharge**<br>a) Hypertension<br>b) Diabetes<br>c) Dyslipidemia	 558 (42.66)<br>223 (17.05)<br>108 (8.25)
**Patient and Caregiver Advice at the time of Discharge**	
Tracheostomy care advice<br>Indicated<br>Not indicated	 195 (14.93)<br>1111 (85.07)
Tracheostomy care advice among indicated<br>a) Yes<br>b) No	 18 (9.23)<br>177 (90.77)
Catheter care advice<br>Indicated<br>Not indicated	 611 (46.68)<br>698 (53.32)
Catheter care advice among indicated<br>a) Yes<br>b) No	 139 (22.75)<br>472 (77.25)
RT care advice<br>Indicated	 596 (45.57)

Not indicated	712 (54.43)
RT care advice among indicated<br>a) Yes<br>b) No	 171 (28.69)<br>425 (71.31)
Positioning advice<br>Indicated<br>Not indicated	 943 (72.15)<br>364 (27.85)
Positioning advice among indicated<br>a) Yes<br>b) No	 339 (35.95)<br>604 (64.05)
**Medication Advice at the time of discharge**<br>a) Adherence to medication (n=1310)<br>b) Medication dose (n=1307)<br>c) Timing of medication (n=1310)<br>d) Adverse Effects of medication (n=1308)	 497 (37.94)<br>1277 (97.70)<br>1198 (91.45)<br>240 (18.35)
**Caregiver advise**	889 (68.02)
**Follow up advice**<br>a) Follow up advice<br>b) When to follow up<br>c) Whom to follow up<br>d) Where to follow up	 1137 (86.86)<br>1138 (86.94)<br>818 (62.44)<br>1124 (85.87)

N (%)- Number of participants (%), LAMA-Leave against medical advice, TOAST- Trial of ORG 10172 in Acute Stroke Treatment, RT-Ryle's Tube

Supplementary Table 2: Between-Site Variation for Door-to-CT Time and Rates of Intravenous Thrombolysis (IVT) use

		Intravenous Thrombolysis (IVT)	
**Collaborating Sites**	**Door-to-CT Time (in min) Median (IQR)**	**Tissue Plasminogen Activator (TPA) n (%)**	**Tenecteplase (TNK) n (%)**
1	74 (26-135)	0 (0)	8 (100)
2	73 (42-148)	6 (60)	4 (40)
3	109 (28-113)	16 (100)	0 (0)
4	36 (32-158)	4 (66.7)	2 (33.3)
5	122 (85-770)	0 (0)	0 (0)
6	45 (51-369)	0 (0)	0 (0)
7	103 (72-787)	0 (0)	0 (0)
8	68 (80-307.5)	0 (0)	1 (100)
9	40 (40-199)	1 (100)	0 (0)
10	23 (60-405)	1 (100)	0 (0)
11	76 (45-120)	3 (100)	0 (0)
12	69 (55-274)	1 (100)	0 (0)

13	26 (16-39)	20 (100)	0 (0)
14	103 (35-89)	0 (0)	0 (0)
15	38 (75-673)	2 (50)	2 (50)
16	36 (88.5-695)	0 (0)	0 (0)
17	153 (43-184)	0 (0)	0 (0)
18	21 (113-350)	0 (0)	0 (0)
19	64 (89.5-410.5)	0 (0)	1 (100)
20	47 (65-555)	0 (0)	1 (100)
21	26 (51-308)	0 (0)	1 (100)
22	12 (58.5-442.5)	0 (0)	0 (0)
23	42 (112-404)	0 (0)	1 (100)

Appendix 1: Case Record Form Checklist

Section A: Admission Baseline Details

- Record ID
- Age (in years)
- Sex
- Date of Stroke
- Time of onset recorded in the file
    - Yes
    - No
- Category of stroke patient based on time from stroke onset
    - Onset within 24 hours
    - 24-48 hours of onset
    - 48-72 hours of onset
- Whether NIHSS recorded at admission
    - Yes
    - No
- Whether Pulse measured
    - Yes
    - No
- Whether Blood Pressure (BP) measured
    - Yes
    - No
- Whether Blood Sugar measured

- Yes
- No

- CT at admission to study facility
    - Yes
    - No
    - Not done as already done
- Type of Stroke
    - Ischemic
    - ICH
    - CVT
    - CT not done
- Hypertension
    - Yes
    - No
- Diabetes Mellitus
    - Yes
    - No
- Smoking
    - No
    - Current
    - Past
- Coronary Artery Disease (CAD)
    - Yes

- No

- Rheumatic Heart Disease (RHD)
    - Yes
    - No
- Atrial Fibrillation
    - Yes
    - No
- Alcohol Intake
    - Yes
    - No
- Dyslipidemia
    - Yes
    - No
- Previous history of stroke
    - Yes
    - No
- Family history of Stroke
    - Yes
    - No

Section B: Thrombolysis and Thrombectomy Data

- Is patient eligible for thrombolysis
    - Yes
    - No

- If not eligible for thrombolysis then reason
    - ICH
    - CVT
    - Not in window period
    - Other contraindications
    - Thrombolysed outside
- Did the eligible patient receive thrombolysis
    - Yes
    - No
- If eligible and not thrombolysed reason
    - Thrombolysis not available
    - Thrombolysis not affordable
    - Thrombolysis available but not given in hospital
    - Delay after admission
    - Refused
- Thrombolysis Type
    - TPA
    - TNK
- Time of Thrombolysis
- Onset to needle time (minutes)
- CT scanner to needle time (minutes)
- Door to needle time (minutes)
- Endovascular Treatment

- Yes
- No

- If Endovascular No, then reason
    - No LVO
    - EVT not available
    - EVT not affordable
    - EVT available but not used
    - Not eligible (time window)
    - Not eligible (Contraindications)
    - Not indicated (Non-Ischemic stroke)
    - Refused

Section C: Laboratory Details

- CTA done at admission site
    - Yes
    - No
    - Not available
    - Not indicated
- MRA study site
    - Yes
    - No
    - Not available
    - Not indicated
- Doppler neck vessels

- Yes
- No
- Not available
- Not indicated

Section D: In hospital details at 24 Hours, 24-48 Hours and 48-72 Hours, separately

- Whether Glasgow coma scale assessed
    - Yes
    - No
- Blood pressure monitoring
    - Yes
    - No
- Blood sugar monitoring
    - Yes
    - No
- Temperature monitoring once daily
    - Yes
    - No
- Whether DVT prophylaxis provided
    - Yes
    - No
    - Not indicated
- If DVT prophylaxis provided, then type
    - Heprain/LMWH

- Compression device
- Both

- Whether swallow assessment done
  - Yes
  - No
  - Not indicated
- Whether appropriate positioning done
  - Yes
  - No
  - Not indicated
- Physio consultation and visit by physiotherapist
  - Yes
  - No
  - Not indicated
- Whether Air Mattress is provided
  - Yes
  - No
  - Not indicated
  - Not available
- Caregiver advice given
  - Yes
  - No
- In hospital complication recorded

- Yes
- No

Section E: Discharge Details

- Patient survived
  - Yes
  - No
- Patient Discharged/LAMA
- In hospital complications
  - Yes
  - No
- TOAST mentioned in discharge summary
  - Yes
  - No
  - Not indicated
- TOAST subtype
  - Large artery
  - Small vessel
  - Cardioembolic
  - Other determined
  - Undetermined
- Risk factor advise in discharge summary
  - Hypertension (Yes/No)
  - Diabetes Mellitus (Yes/No)

    - Dyslipidemia (Yes/No)

- Tracheostomy (TT) care advise in discharge summary
    - Yes
    - No
    - Not indicated
- Catheter care advise in discharge summary
    - Yes
    - No
    - Not indicated
- Ryle's tube (RT) care advise in discharge summary
    - Yes
    - No
    - Not indicated
- Positioning care advise in discharge summary
    - Yes
    - No
    - Not indicated
- Medication mentioned optimally
    - Yes
    - No
- Dose mentioned in discharge summary
    - Yes
    - No

- Adverse effect warning in discharge summary
    - Yes
    - No
- Timing of medication in discharge summary
    - Yes
    - No
- Adherence advise in discharge summary
    - Yes
    - No
- Caregiver advise in discharge summary
    - Yes
    - No
- Follow up advise documented
    - Yes
    - No
- When to Follow up in discharge summary
    - Yes
    - No
- Whom to follow up with in discharge summary
    - Yes
    - No
- Where to follow up in discharge summary
    - Yes

- No

Section F: Follow-up Details

- Date of 3 months follow-up
- Modified Rankin Score (mRs) at 3 months: 0-6
